# Patient with CHARGE syndrome

**DOI:** 10.11604/pamj.2018.31.51.7029

**Published:** 2018-09-24

**Authors:** Abdelhakim El Yajouri, Chafiq Mahraoui

**Affiliations:** 1 Pneumo-allergology unit of Rabat Children Hospital, Faculty of Medicine, Mohammed V University Rabat, Morocco

**Keywords:** CHARGE syndrome, health care consumption, long-term conditions

## Abstract

A two-years-old boy presenting multiple anomaly syndrome (congenital heart disease, Tetralogy of Fallot, bilateral ear anomalies, and micropenis) was consulted in the Pneumo-allergology unit of Rabat children hospital in Morocco. It was ruled out the possibility of multiple deformities caused by genomic imbalances. The patient was then clinically considered to have CHARGE syndrome, an autosomal dominant multi-system disorder involving defects in multiple organs.

## Introduction

CHARGE syndrome is an autosomal dominant multisystem disorder involving coloboma, heart defects, choanal atresia, delayed growth and development, genital hypoplasia, ear anomalies and/or deafness [1]. In 1998, Blake *et al.* established reformative diagnostic criteria for CHARGE syndrome comprising of major and minor criteria. Major criteria include coloboma, choanal atresia, ear anomalies/ deafness and cranial nerve dysfunction; while minor criteria include heart defects, genital hypoplasia, growth deficiency, developmental delay, tracheoesophageal fistula, orofacial cleft, and distinctive facial appearance [2].

## Patient and observation

**Figure 1:**
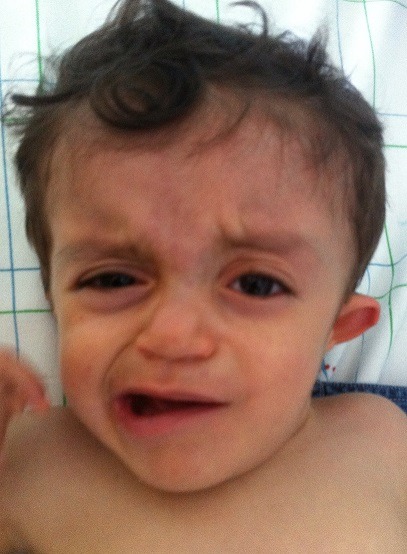
facial features in CHARGE syndrome

The patient is 2-year-old male, first born child of non-consanguineous Moroccan healthy parents with an ordinary family history, after uncomplicated pregnancy and delivery. After birth, the infant had been suffering from recurrent respiratory tract infections and frequently admitted to the hospital. Subsequent examinations revealed multiple malformations such as unique facial features (Figure 1), Tetralogy of Fallot, bilateral ear anomalies, and micropenis. Based on the above observations, he was suspected to have CHARGE syndrome.

## Discussion

Upon the first admission to the hospital, the patient presented unique facial features, congenital heart disease, genital hypoplasia and retarded growth/development [2]. Typical and atypical symptoms were shown in (Table 1). As evidence of the diagnosis, the patient presented: coloboma: a left-sided iris coloboma was identified, while both visual acuity and visual field were not affected; heart defects: ultrasound cardiogram showed Tetralogy of Fallot; choanal atresia: nasopharyngoscopy showed choanal atresia caused by abnormal soft tissue, with half of the choanae being obstructed by nasopharyngeal adenoids hypertrophy; growth retardation; genital hypoplasia: External genital examination showed micropenis (about 2.2 cm), and ultrasound revealed bilateral inguinal cryptorchidism, and ear anomalies: auditory brainstem response test showed bilateral hearing loss. Children with CHARGE syndrome require intensive medical management as well as numerous surgical interventions. The most common neonatal emergencies in CHARGE syndrome involve cyanosis due to bilateral posterior choanal atresia and/or congenital heart defects, or the less common presentation of trachea-esophageal fistula [3]. All patients suspected of having CHARGE syndrome should have a cardiology consultation. If the infant has restrictive pulmonary blood flow and is dependent on a patent ductus arteriosus, the administration of prostaglandin to maintain ductal patency may be lifesaving. Some children require tracheostomy to manage chronic airway problems and/or gastro-oesophageal reflux and aspiration. Children with CHARGE syndrome require aggressive medical management of their feeding difficulties, often needing gastrostomy and jejunostomy feeding tubes. Gastro-oesophageal fundoplication may be required for GER that does not respond to medical management. As intubation can be difficult in children with CHARGE syndrome, a pediatric anesthesiologist or pediatric otolaryngologist should be present for planned surgical procedures [3]. Any infant suspected of having CHARGE syndrome should have a complete eye examination by an ophthalmologist, with follow-up every three to six months there- after, depending on the eye involvement. Photophobia is often a significant problem that can be ameliorated with tinted spectacles or by wearing a cap or visor with a dark brim. In the presence of facial palsy, patients should avoid corneal scarring by using artificial tears [4]. Hearing aids should be used as soon as hearing loss is documented. Frequent remolding of the earpieces is necessary as the ear canals can be initially very small and ear cartilage may be insufficient to support a hearing aid. Cochlear implantations have been successfully performed in CHARGE syndrome patients. Children with CHARGE syndrome who undergo cochlear implantation should be allowed to continue with their sign language in parallel with their expressive speech training [4]. In terms of endocrine issues, sex steroid therapy has been used for penile growth and descent of testes in males with CHARGE syndrome. The main use for testosterone is for delayed and incomplete male puberty during adolescence. Females often require hormone replacement at puberty [5]. Sex hormone replacement is also indicated for prevention of osteoporosis [6].

**Table 1:** typical and atypical symptoms of the patient in this case

Diagnostic criteria of CHARGE	Symptoms of the patient in this case
**MAJOR**	
Ocular coloboma	+
Choanal atresia	+
Characteristic external ear anomaly, or middle ear malformations or mixed deafness	+
Cranial nerve dysfunction	+
**MINOR**	
Congenital cardiovascular malformations	+
Tracheoesophageal defect	+
Genital hypoplasia/delayed pubertal development	+
Developmental delay	-
Growth retardation	+
Characteristic face	+
**Atypical**	
Macrocrany	+

## Conclusion

The present findings highlight CHARGE syndrome as a highly complex medical condition, leading to a significant consumption of health care during the first year in life, as well as a considerable amount of different health care contacts.

## Competing interests

The authors declare no competing interests.

## Authors’ contributions

All authors have read and agreed to the final version of this manuscript and have equally contributed to its content and to the management of the case.

## References

[1] Aramaki M, Udaka T, Kosaki R, Makita Y, Okamoto N, Yoshihashi H, Oki H, Nanao K, Moriyama N, Oku S, Hasegawa T, Takahashi T, Fukushima Y, Kawame H, Kosaki K (2006). Phenotypic spectrum of CHARGE syndrome with CHD7 mutations. J Pediatr.

[2] Bajpai R, Chen DA, Rada-Iglesias A, Zhang J, Xiong Y, Helms J, Chang CP, Zhao Y, Swigut T, Wysocka J (2010). CHD7 cooperates with PBAF to control multipotent neural crest formation. Nature.

[3] Blake KD, Davenport SLH, Hall BD, Hefner MA, Pagon RA, Williams MS, Lin AE, Graham JM (1998). CHARGE association: an update and review for the primary pediatrician. Clin Pediatr (Phila).

[4] Thelin JW, Fussner JC (2005). Factors related to the development of communications in CHARGE syndrome. Am J Med Genet A.

[5] Forward K, Cummings E, Blake K (2005). Bone health in adolescents and adults with CHARGE syndrome.

[6] Lalani SR, Safiullah AM, Fernbach SD, Harutyunyan KG, Thaller C, Peterson LE, McPherson JD, Gibbs RA, White LD, Hefner M, Davenport SL, Graham JM, Bacino CA, Glass NL, Towbin JA, Craigen WJ, Neish SR, Lin AE, Belmont JW (2006). Spectrum of CHD7 Mutations in 110 Individuals with CHARGE Syndrome and Genotype-Phenotype Correlation. Am J Hum Genet.

